# An observational study on non-communicable disease risk factors among healthcare workers in high-stress environments

**DOI:** 10.3389/fpubh.2025.1609034

**Published:** 2025-05-26

**Authors:** Alessandra Pirrello, Daniele Giacomo Mancuso, Chiara Pace, Angelo Immordino, Virginia Meli, Fabio Tramuto, Alessandra Casuccio, Palmira Immordino

**Affiliations:** ^1^Department of Health Promotion, Mother and Child Care, Internal Medicine and Medical Specialties, University of Palermo, Palermo, Italy; ^2^University Hospital AOUP "Paolo Giaccone", Palermo, Italy

**Keywords:** non-communicable diseases, healthcare workers, lifestyle risk factors, health promotion programs, occupational health

## Abstract

**Introduction:**

Non-communicable diseases (NCDs) account for 74% of global mortality and place significant socioeconomic burdens on healthcare systems. Despite their knowledge and awareness, healthcare workers (HCWs) often struggle to adopt preventive measures due to demanding work conditions and high-stress environments.

**Methods:**

This observational study aimed to evaluate the prevalence of NCD risk factors among HCWs at the University Hospital “Paolo Giaccone” in Palermo, Italy. An online questionnaire, based on the WHO’s STEPS approach, was administered to 390 HCWs. Data from 273 responses were analyzed using SPSS software.

**Results:**

The sample comprised 57.9% women, predominantly nurses (35.9%).

**Discussion:**

Key findings revealed that 56.8% consumed alcohol, 42.1% used tobacco, and 86.1% frequently skipped meals due to work. Lifestyle factors, such as fruit and vegetable consumption, salt intake, and physical activity, were assessed alongside metabolic risk factors like blood pressure, glycemia, and triglycerides. Despite their expertise in NCD prevention, HCWs often failed to implement healthy behaviors. While evening shift work showed limited correlation with lifestyle changes, results underscored the need for targeted health promotion programs for HCWs. Healthcare institutions should actively support their workforce in adopting healthier lifestyles to mitigate NCD risks and improve public health outcomes.

## 1 Introduction

Non-communicable diseases (NCDs), are long-lasting conditions not caused by infectious agents triggered by various risk factors, and marked by slow progression and/gradual deterioration of organ or tissue function. To date, NCDs represent the leading cause of death and disability worldwide. The estimates produced by the World Health Organization (1), called Global Health Estimates (GHE) between 2000 and 2019 speak clearly: NCDs with 74% of total deaths (72% in males and 75% in females) are the main cause of death worldwide and, in Italy, it has been estimated that they were the main cause of mortality in 91% of cases (90% in males and 91% in females) (2).

The most widespread NCDs globally are currently grouped into four main groups: cardiovascular diseases, tumors, chronic respiratory diseases and diabetes; together they constitute almost 80% of all causes of death globally (3). In the population of individuals aged 30 to 69 years, nearly half (49%) of NCDs are considered preventable through appropriate public health interventions, lifestyle modifications, and access to timely healthcare. This staggering statistic translates to approximately 2.4 million deaths each year that could be avoided, along with the prevention of 93.8 million disability-adjusted life-years (DALYs)—a measure reflecting years of healthy life lost due to illness or premature mortality (4).

In Italy, NCDs impact approximately 24 million individuals, significantly influencing both life expectancy and quality of life. While these conditions can affect people at all life stages, they are most prevalent among the older adult—over 85% of individuals aged 75 and above are affected—and among women, particularly those over the age of 55 (5). Data observed at global and national level align with those recorded in Sicily where, in 2020, the statistics provided by ISTAT (6) confirm 19,078 deaths from cardiovascular diseases, 13,067 from tumor pathologies, 2,053 from chronic diseases of the respiratory system, 3,014 for diabetes mellitus.

One of the main characteristics of NCDs is, undoubtedly, the etiological multifactorial nature. Modifiable risk factors include tobacco and alcohol consumption, lack of physical activity, overweight and obesity, increased fat and salt consumption, low fruit and vegetable consumption, increased blood pressure, increased glucose levels and cholesterol that predispose to the development of NCD (1).

Healthcare workers (HCWs)are not exempt from the risk of NCDs and represent a critical population for examining individual health behaviors. This is due to their heightened awareness of health-related choices compared to the general population and their pivotal role in advising patients while modeling and promoting healthy lifestyles and behaviors. The World Health Organization (WHO) broadly defines HCWs as all people engaged in actions whose primary intent is to enhance health. This therefore includes doctors, nurses, obstetricians, public health professionals, laboratory, personal care workers, community workers, paramedical staff, as well as non-medical staff who carry out assistance-related activities, such as support staff ranging from rescue drivers to company managers (7).

Those who are employed in the provision of healthcare are exposed to numerous risks to their health daily, including biological risk, chemical risk, physical risk, ergonomic risk and psychosocial risk (8).

All the factors listed above are already widely known, studied and find their recognition in the implementation of mandatory preventive strategies, not only by the employer, but primarily for the worker himself and included in the current legislation which corresponds to the D.lgs. 81/08, the Consolidated Law on the health and safety of workers (9).

The healthcare sector is among the most dangerous working environment from the point of view of health and well-being with less than 1/6 of countries that have issued a national-level policy for the health and safety of workers in the healthcare sector (10), with an ever-increasing number of HCWs contracting infections, occupational diseases and injuries every day at work, who are subjected to strong psychological and emotional stress which can degenerate into burnout. All these factors if not timely identified and managed, may cause significant distress and increased risk for the development of long-term consequences.

This situation could lead to absenteeism, long periods of illness, personal and professional dissatisfaction, high turnover of operators, instability in the work organization, poor relationships between colleagues and numerous other problems that significantly affect the quality of the work environment and its general performance (11).

As far as NCDs and related risk factors are concerned, although they are increasingly relevant and in-depth topics in the literature, there is still very little evidence that focuses on individual work environments, particularly healthcare and health professionals. Healthcare workers face unique occupational challenges, including high levels of stress, long working hours, irregular schedules, and exposure to demanding clinical or administrative conditions. These factors may significantly influence health behaviors and amplify certain risk factors such as physical inactivity, unhealthy diets, smoking, or stress-related conditions (12).

Understanding the interplay between occupational settings and individual health choices is crucial, as healthcare workers are not only at risk themselves but also serve as role models and advisors for patients.

The objective of this study is to assess among HCWs, the prevalence of risk factors contributing to the development of NCDs, while also exploring the role of the work environment in shaping these risks. Moreover, this study aims to identify workplace dynamics that may contribute to the risk of NCDs, thereby providing insights into targeted interventions to improve occupational health and promote healthier behaviors in this critical workforce.

## 2 Materials and methods

An analytical cross-sectional survey-based study was carried out from 20th June until 31st July 2023. The study included HCWs of the University Hospital “Paolo Giaccone.” – Palermo (Italy) of both sexes, aged between 18 and 70 years, working at the University Hospital “P. Giaccone,” who voluntarily decided to complete the administered questionnaire. Data collection was conducted using a structured questionnaire designed to assess key variables related to NCDs and associated risk factors. The questionnaire was distributed digitally using the “Google Form” platform, ensuring accessibility and ease of response for participants. The structured format of the questionnaire enabled the systematic collection of data, allowing for the evaluation of health behaviors, risk factors, and the potential influence of occupational environments on the prevalence of NCD-related risk factors among healthcare workers.

The questions were extrapolated from the questionnaire used to implement the WHO “STEPS” approach (STEPwise approach to NCD risk factor surveillance).

The WHO STEPwise Approach to Surveillance (STEPS) is an internationally comparable, standardized and integrated surveillance tool through which countries can collect, analyze and disseminate key information on non-communicable diseases (13).

The questionnaire comprises three main sections, beginning with an introductory segment that clearly outlines the objectives and methodology of the study in a concise and comprehensive manner. This section also includes essential information regarding data privacy and the handling of personal and sensitive data, in compliance with the provisions of D. lgs 101/2018. This introductory section also informed participants on the anonymity of the data collection and analysis as well as the possibility that the research results may be published in medical journals or presented at conferences to advance scientific knowledge.

The first section focuses on collecting basic demographic and personal information. The second section is structured into thematic subsections and contains a total of 26 multiple-choice questions designed to assess common risk factors for non-communicable diseases (NCDs). These include queries on alcohol and tobacco use, dietary habits, salt intake, physical activity levels, vital signs, and any prior diagnoses of risk factors associated with chronic conditions.

We evaluated two groups of main risk factors: behavioral and metabolic.

The assessment of the key variables was conducted as follows:

- Alcohol consumption was estimated based on the intake of alcoholic beverages in the previous 30 days and based on the frequency of intake in the given period.
- Tobacco use was calculated on those who declared having smoked in the last 30 days and based on the frequency of smoking cigarettes and similar products.
- For the “eating habits” variable, was asked about the weekly frequency of “eating fruit and vegetable meals” and the “habit of consuming meals related to working hours.”
- The consumption of salt and products with a high sodium content was estimated based on the habitual addition of salt to food and the frequency with which this occurs.
- For physical activity, work-related information was collected, in terms of 3 factors: a consistent and/or moderate increase in heart rate and/or breathing; activity practiced habitually over a week for at least 20–30 min; the methods of traveling for short distances and a personal opinion on the perception of one’s own level of physical activity.
- For risk factors, questions regarding the variables “measurement of blood pressure, blood sugar and cholesterol/triglyceridemia” were included with the help of a qualified operator.
- For chronic diseases, previous history of arterial hypertension, diabetes, hypercholesterolemia/hypertriglyceridemia and adherence to therapy in relation to working hours were evaluated.

The final segment comprises two additional concise multiple-choice subsections aimed at evaluating potential behavioral changes that could either reduce or increase the risk of developing NCDs. This structured approach ensures a comprehensive evaluation of participants’ health behaviors and their potential modifiability in relation to NCD prevention. The questionnaire was proposed to all HCWs and was completed voluntarily and anonymously, involving almost all levels of the organization outlined by Henry Mintzberg, except for the strategic top management. Nurses and doctors represented the largest sample followed by administrative staff and the IT staff. To ensure the widest dissemination, the invitation to fill up the questionnaire was published on the Intranet of the University Hospital, prior to authorization from the “Palermo 1” ethics Committee.

### 2.1 Statistical analysis

The required minimum sample size for this investigation was calculated using Epi InfoTM version 7.2.5 (CDC, Atlanta, GA, USA, 2021) and was 335 subjects with a potential drop-out of 25%. The sample size was calculated to provide 80% power with *α* = 0.05.

The data are expressed as a mean ±SD, or frequency (%). Differences between gender and age were assessed by a chi-square test or Fisher’s exact test, as needed for categorical variables, and by univariate analysis of variance (ANOVA) for parametric variables. Characteristics of the habits and lifestyle of the HCWs (dependent variables) correlated with evening/night shift work activity (independent variable) were evaluated by univariable (Crude OR) and multivariable (Adjusted OR) regression analysis.

The data were analysed using IBM SPSS Software version 24 (IBM Corp., Armonk, NY, USA). All *p* values were two-tailed, and *p* ≤ 0.05 was considered to indicate statistical significance.

## 3 Results

A total of 273 healthcare workers (around 80% of the study population) at the University Hospital “Paolo Giaccone” filled the questionnaire, out of approximately 335 HCWs to whom the questionnaire was proposed.

The sample obtained was then classified, using the Sturges rule, within the following age classes:

- I class: 23–29 years.
- II class: 30–50 years.
- II class: 51–64 years.
- IV class: 65–70 years.

Table 1 reports the characteristics of the study population, represented mostly by females (57.9%), and with average age 47.0 (±11.9) years. The most represented age group was the 53–58 years (17.9%). The various health professionals were represented as follows: nurses (35.9%), physicians/residents (25.6%), healthcare support workers (9.5%), health technicians (9.2%), other HCWs (19.8%) More than a half (55.7%) of the respondents reported working evening/night shifts.

**Table 1:** Characteristics of the study population.

	*N* (%)
Age classes	
23–28	19 (7.0)
29–34	33 (12.1)
35–40	41 (15.0)
41–46	32 (11.7)
47–52	43 (15.8)
53–58	49 (17.9)
59–64	42 (15.4)
65–70	14 (5.1)
Gender	
Male	115 (42.1)
Female	158 (57.9)
Work	
Nurses	98 (35.9)
Physicians/residents	70 (25.6)
Healthcare support workers	26 (9.5)
Health technicians	25 (9.2)
Others	54 (19.8)
Shift work	
Yes	152 (55.7)
No	121 (44.3)

Table 2 summarizes the results related to the habits and lifestyle of those interviewed, investigating the prevalence of the various behavioral risk factors. More than half of the respondents (56.8%) reported having consumed alcohol in the last 30 days, and the most frequent group (38.7%) reported consumption 1–2 times per week. No differences emerged between sexes in alcohol consumption (*p* = 0.09), while younger individuals tended to consume significantly more alcohol (*p* < 0.0005).

**Table 2:** Characteristics of the habits and lifestyle of the HCWs interviewed.

Alcohol and tobacco consumption	
Have you drink alcoholic beverages in the 30 days after?	
Yes	155 (56.8%)
No	118 (43.2%)
If yes, how often	
Every day	3 (1.9%)
5–6 times a week	4 (2.6%)
3–4 times a week	10 (6.5%)
1–2 times a week	60 (38.7%)
1–3 times a month	49 (31.6%)
Less than 1 a month	29 (18.7%)
Have you ever smoked/do you smoke cigarettes or other products with tobacco?	
Yes	115 (42.1%)
No	158 (57.9%)
If yes, how many cigarettes do you smoke daily?	
More than 20	9 (7.8%)
11–20	30 (26.1%)
6–10	35 (30.4%)
1–5	41 (35.7%)
How often do you eat fruits?	
Every day	165 (60.4%)
5–6 times a week	17 (6.2%)
3–4 times a week	35 (12.8%)
1–2 times a week	32 (11.7%)
Less than 1 a week	24 (8.8%)
How often do you eat vegetables?	
Every day	103 (37.7%)
5–6 times a week	37 (13.6%)
3–4 times a week	66 (24.2%)
1–2 times a week	48 (17.6%)
Less than 1 a week	19 (7.0%)
How often do you add salt or high-sodium condiments (e.g., bouillon cubes, ketchup, soy sauce, etc.) to your meals?	
Always	24 (8.8%)
Often	41 (15.0%)
Sometimes	82 (30.0%)
Rarely	93 (34.1%)
Never	33 (12.1%)
How often do you consume processed foods with a high salt content? (e.g., packaged savory snacks, cheese, processed meat, fast food, canned foods).	
Always	7 (2.6%)
Often	50 (18.3%)
Sometimes	124 (45.4%)
Rarely	80 (29.3%)
Never	12 (4.4%)
Have you ever skipped a meal because of your work shifts?	
Yes	235 (86.1%)
No	38 (13.9%)
If yes, how often?	
Always	12 (5.1%)
Often	80 (34.0%)
Sometimes	106 (45.1%)
Rarely	37 (15.8%)
Does your job involve high-intensity physical activities that can cause a significant increase in heart rate and/or breathing?	
Yes	78 (28.6%)
No	195 (71.4%)
Does your job include moderate intensity physical activities that may cause modest changes in heart rate and/or breathing?	
Yes	153 (56%)
No	120 (44%)
During the week, do you practice a high/medium intensity sporting or physical activity or hobby for at least 20–30 min?	
Yes	115 (42.1%)
No	158 (57.9%)
If yes, how often?	
Every day	6 (5.2%)
5–6 times a week	5 (4.3%)
3–4 times a week	38 (33.0%)
1–2 times a week	58 (50.5%)
Less than 1 a week	8 (7.0%)

To evaluate smoking habits, the questionnaire investigated those who smoked tobacco products at least once, resulting in 42.1% positive response. The most represented group was those who smoked fewer than 5 (35.7%) and 10 (30.4%) cigarettes per day. No significant differences emerged between sexes in smoking habits (*p* = 0.258); smokers were also more frequent in the younger age group (*p* = 0.012).

Overall, 66.6% (*N* = 182) and 51.3% (*N* = 140) of respondents reported consuming fruit and vegetables daily or 5–6 times per week, respectively, with a higher fruit consumption among males (*p* = 0.030) and a higher vegetable consumption among females (*p* < 0.0005). Older individuals reported consuming more fruits (*p* < 0.0005), while no age differences were observed in vegetable consumption.

The consumption of salt and the intake of processed foods with a high sodium content appeared to be moderate in both sexes and for all age groups.

It also emerged that 235 interviewed (86.1%) had skipped (often/sometimes) a meal due to working hours.

Regarding physical activity in the workplace, 71.4% versus 28.6% responded that their work does not involve high intensity activities that can cause a significant increase in heart rate and/or breathing; while 56% versus 44% said that their work involves moderate intensity activity, which can cause modest changes in heart rate and/or breathing.

The questionnaire included also behavioral questions such as the mode of travel for short distances. The majority of those interviewed, 44.7%, seemed to prefer moving on foot, while the second most used means of transport are the car and public transport (37%) and, in modest percentages, motor vehicles (12.8%).

Physical activity was further investigated, and it was found that only 42.1% practiced a high/medium intensity sporting, physical activity or hobby for at least 20–30 min, however, with a weekly frequency of 1–2 times or less.

Table 3 includes the results related to the prevalence of metabolic risk factors. The presence of any NCDs in the anamnesis and the relationship with any related therapy were also investigated.

**Table 3:** Characteristics of the NCDs and metabolic risk factors of the HCWs interviewed.

Non-communicable disease	Yes	No
Have you ever measured your blood pressure, at least once, with the help of a professional?	259 (94.9%)	14 (5.1%)
Have you ever had blood pressure readings above average and/or been diagnosed with hypertension?	86 (31.5%)	187 (68.5%)
Have you ever measured your glycemic values, at least once, with the help of a professional?	247 (90.5%)	26 (9.5%)
Have you ever had blood sugar levels above average or been diagnosed with diabetes?	40 (14.7%)	233 (85.3%)
Have you ever measured, at least once, your cholesterol levels (HDL, LDL) and/or triglycerides?	258 (94.5%)	15 (5.5%)
Have you ever experienced high cholesterol and/or triglycerides?	134 (49.1%)	139 (50.9%)
If you have ever been diagnosed with a chronic disease (e.g., hypertension, diabetes) do you regularly take medications for your condition?	84 (30.8%)	156 (57.2%)

From a univariate analysis, evening shift resulted significantly associated with younger age (*p* < 0.0005), alcohol consumption (*p* = 0.043), impact of shifts on the quality/quantity of meals (*p* < 0.0005), salt consumption (*p* = 0.002), and inversely correlated with the possibility of high blood pressure (*p* = 0.040), high blood sugar (*p* = 0.005), and high cholesterol (*p* = 0.036).

In a multivariable model, the evening shift resulted significantly associated with younger age, salt consumption, and the impact of shifts on the quality of meals, while hyperglycemia was inversely associated with evening shifts.

For the hypertension factor, 94.9% of those interviewed had their blood pressure measured at least once; furthermore, 31.5% had received a diagnosis of hypertension. 22% of women and 44% of men had values above the average and/or received a diagnosis of hypertension (*p* < 0.0005). The diagnosis of hypertension was associated with an older age (*p* < 0.0005), and the frequency increases with age, reaching 88% among those over 55.

In terms of diabetes secondary prevention, 90.5% of those interviewed had their blood sugar measured with the help of a professional. Overall, 14.7% of the respondents reported elevated blood glucose levels without sex differences but associated with a higher average age (*p* = 0.006), with 80% of the cases occurring in individuals over 40 years of age. For the metabolic risk factor “dyslipidemia,” 94.5% carried out this measurement with the help of a professional. Among those who measured hypercholesterolemia and triglyceridemia, 49.1% of both women and men found values above the physiological threshold, with a significant association with a higher average age (*p* < 0.0005) and 68% of the cases occurring in individuals over 45 years of age. Finally, respondents diagnosed with NCD were asked whether they were able to take medications regularly for their condition, and only 30.8% said they took medications regularly.

Ultimately, the attitude of the interviewees toward a possible change of their lifestyle in order to improve their health was observed and the results were the following: 26.4% thought about quitting smoking, but only 13.9% actually implemented this behavior; 33.7% thought about increasing their weekly fruit and vegetable intake and 27.5% implemented this decision; again, 27.5, 42.1 and 38.8% thought of reducing the daily intake of salt, fat and sugar in their diet, respectively, and 24.2, 38.5 and 34.1% paid attention to implementing this indication.

Finally, for the physical activity factor, 57.5% thought at least once about starting or carrying out more physical activity and 62.6% thought it appropriate to pay attention to achieving and maintaining an optimal weight, but only 40.7 and 52% carried out this choice (Table 4).

**Table 4:** Factors associated with evening/night shift work activity at uni (Crude OR) and multivariable (Adjusted OR) regression analysis (95% CI: 95% confidence interval).

	Evening/night shift work activity			
	Crude OR (95% CI)	*p*-value	Adj-OR (95% CI)	*p*-value
Age				
	Ref.	<0.0005	Ref.	<0.0005
	0.943 (0.922–0.964)		0.947 (0.920–0.974)	
Sex				
Female	Ref.	0.137	Ref.	0.224
Male	0.693 (0.427–1.124)		0.689 (0.378–1.257)	
Fruit consumption (5–6 times a week or more)				
Yes	Ref.	0.169	Ref.	0.781
No	1.433 (0.858–2.394)		0.906 (0.452–1.817)	
Vegetable consumption (5–6 times a week or more)				
Yes	Ref.	0.990	Ref.	0.856
No	0.997 (0.618–1.608)		0.943 (0.500–1.780)	
Consumed alcohol in the last 30 days (5–6 times a week or more)				
No	Ref.	0.043	Ref.	0.509
Yes	1.648 (1.016–2.673)		1.211 (0.686–2.139)	
Smoking tobacco products (10 cigarettes or more)				
No	Ref.	0.108	Ref.	0.309
Yes	1.494 (0.916–2.436)		1.338 (0.7642.343)	
Salt consumption (Often/sometimes)				
No	Ref.	0.002	Ref.	0.040
Yes	1.538 (1.177–2.010)		1.352 (1.010–1.823)	
Skipped meals (Often/simetimes)				
No	Ref.	0.684	Ref.	0.858
Yes	1.153 (0.580–2.293)		1.073 (0.496–2.320)	
Physical activity (3–4 times a week)				
Yes	Ref.	0.627	Ref.	0.515
No	0.887 (0.546–1.439)		1.216 (0.676–2.188)	
Diagnosis of hypertension				
No	Ref.	0.040	Ref.	0.238
Yes	0.582 (0.348–0.974)		1.489 (0.768–2.888)	
Hyperglycemia				
No	Ref.	0.005	Ref.	0.030
Yes	0.371 (0.184–0.747)		0.405 (0.179–0.918)	
Hypercholesterolemia/hypertriglyceridemia				
No	Ref.	0.036	Ref.	0.435
Yes	0.598 (0.370–0.968)		0.802 (0.461–1.395)	
On a scale of 1–5, do you think work hours affect the quality of your meals?				
No	Ref.	<0.0005	Ref.	<0.0005
Yes	1.755 (1.403–2.196)		1.682 (1.300–2.175)	

## 4 Discussion

The aim of our study was to determine the prevalence of NCD risk factors and their association with shift work among HCWs at a University teaching hospital.

Our study reveals a complex interplay between lifestyle habits, occupational patterns, and NCD risk. The findings show that over half of respondents reported alcohol consumption in the past 30 days (56.8%) and 42.1% reported a history of smoking, with both behaviors significantly more common among younger individuals. Although the majority consumed fruits daily or almost daily, and 51.3% reported similar frequency for vegetables, only a minority engaged in regular physical activity of moderate-to-high intensity (42.1%), and a striking 86.1% reported skipping meals due to work-related constraints. In terms of metabolic risk factors, 31.5% had been diagnosed with hypertension (with prevalence increasing to 88% in those over 55), 14.7% had elevated blood glucose (primarily over age 40), and 49.1% reported dyslipidemia, with a strong age association (68% over age 45). Despite these health risks, adherence to medical therapy was low, with only 30.8% of those diagnosed with an NCD reporting regular medication intake. Interestingly, evening shift work was associated with greater behavioral risks (e.g., alcohol and salt consumption), but inversely correlated with the presence of hyperglycemia, hypertension, and dyslipidemia. These findings underscore the dual burden of behavioral and occupational factors influencing health among HCWs and highlight both age- and shift-related vulnerabilities that warrant targeted intervention.

Several studies have investigated the development of NCDs in the category of shift workers, yet relatively few have focused specifically on HCWs, despite their high exposure to rotating schedules and occupational stress. A notable Norwegian cross-sectional study (14) involving over 23,000 HCWs found a significant association between shift work and musculoskeletal disorders, particularly affecting the cervical and dorsal-lumbar spine, conditions frequently leading to chronic disabilities. This association was hypothesized to stem from shift work-induced disturbances, which elevate inflammatory markers such as C-reactive protein (CRP). Emerging evidence suggests that these systemic inflammation can mediate the link between irregular work hours and chronic musculoskeletal pain, though the study acknowledged limitations and called for further longitudinal research. Complementing these findings, a Scandinavian cohort study (15) compared two groups of HCWs - those engaged in regular shifts versus those assigned to fixed evening or night shifts.

One year after the baseline survey, individuals working with fixed evening and/or night shifts, exhibited higher risk of obesity, greater tobacco consumption and reduced or absent physical activity during free time, underscoring the multifaceted impact of shift work on behavioral and metabolic risk factors.

Some studies have suggested that rotating shift work may be associated with adaptive lifestyle changes or reduced perceived stress over time. For instance, a cross-sectional study by Chiang et al. (16) investigated the impact of shift patterns on lifestyle and stress among hospital nurses, revealing important associations between work schedules and health-related behaviors. The study found that nurses on fixed day shifts reported significantly lower levels of perceived stress compared to those working rotating shifts. Moreover, among rotating-shift nurses, those on fixed evening or night schedules experienced longer sleep duration than those on variable rotating schedules. Interestingly, longer duration of rotating shift work was positively associated with healthier dietary behavior, improved sleep quality, and reduced perceived stress, although it also correlated with slightly shorter sleep duration. These findings suggest that both the type and consistency of shift schedules influence nurses’ ability to adapt lifestyle habits, with fixed shifts offering potential benefits in mitigating stress and supporting healthier routines.

Also, data from the Sixth European Working Conditions Survey (17) have shown that shift workers, including those in healthcare, are more likely to experience work-related stress, irregular sleep, and poor work–life balance, which are recognized contributors to long-term health risks. However, specific lifestyle-related behaviors were not directly assessed.

Given the scarce evidence regarding the effects of shift work on health-related behaviors, our decision to focus on healthcare workers (HCWs) was further supported by their unique position at the intersection of occupational vulnerability and public health responsibility. Choosing HCWs as our target population was also motivated by their dual role as caregivers and health models. Despite their heightened awareness compared to the general population, many HCWs still struggle to adhere to recommended strategies for NCD prevention, highlighting a critical disconnect between knowledge and practice. This points to the urgent need for institutional health promotion strategies tailored to this population. Policy interventions—such as the integration of physical activity zones within healthcare facilities and structured, protected mealtimes for shift workers—could significantly enhance both individual health outcomes and the broader sustainability of the healthcare system.

While certain risk factors appear to be associated with shift work, our findings suggest that lifestyle behaviors may play a more significant mediating role than work schedules alone. In most of cases, rather than the mere presence of night or evening shifts, it is often the behavioral adaptations individuals make in response to their working routines - such as increased tobacco use or irregular eating patterns - which may or may not lead to the development of cardiovascular and metabolic diseases. For instance, similar observations were made in a cross-sectional study conducted on railway network workers, which found significantly higher levels of total and LDL cholesterol levels among shift workers compared to their day-working counterparts (18). However, the limited number of scientific studies specifically targeting healthcare professionals poses a limitation, underscoring the need for more tailored investigations within this occupational group, to be able to make meaningful comparisons. Our study aimed to address this gap by focusing on healthcare workers (HCWs) as a unique subgroup of shift workers who are simultaneously exposed to occupational strain and elevated health risks. Despite being predominantly younger, those working evening shifts reported compromised dietary patterns, with a substantial proportion (86.1%) indicating they frequently skipped meals due to work demands. Importantly, most respondents reported having undergone objective assessments of key health indicators—including blood pressure, blood glucose, cholesterol, and triglycerides—demonstrating a relatively high level of health awareness. This was further reflected in their self-reported intentions to improve lifestyle behaviors, such as increasing fruit and vegetable intake, reducing salt and fat consumption, and enhancing physical activity levels, even if the actual implementation of these changes remained suboptimal.

Several limitations must be acknowledged. First, the lack of a control group limits causal inference and reduces the ability to generalize findings beyond the study population. While our cross-sectional design provides important insights into current health patterns among HCWs, longitudinal studies with appropriate comparison groups are needed to better understand temporal trends and causal relationships. Second, reliance on self-reported data introduces the potential for recall bias and social desirability bias, particularly in reporting behaviors such as alcohol and tobacco use or adherence to healthy practices. While the use of a structured, anonymous questionnaire may have mitigated some of these concerns, future studies could benefit from integrating objective measurements and clinical assessments. Lastly, while our sample was substantial relative to the population size, it represents a single institution, and broader multi-center studies would strengthen the external validity of the findings. Another limitation of this study is the use of a shortened physical activity assessment tool consisting of questions that did not capture active commuting. This choice was intentional to reduce respondent burden. Additionally, Palermo’s urban setting reduces the relevance of commuting-related physical activity in this population. However, this omission may have led to an underestimation of total physical activity which should be considered when interpreting the findings.

On the other hand, the added value of our study, in addition to being one of the first to be conducted at a University teaching hospital, is to be able to represent not only a stepping stone for developing other studies on the matter, but also to encourage policy makers to understand that the best strategy to safeguard the national healthcare system is the prevention of NCDs in every work, healthcare sector and not.

The findings of this study carry several practical implications for occupational health policy and preventive strategies in healthcare settings. The high prevalence of modifiable risk behaviors—such as alcohol consumption, irregular eating patterns, and physical inactivity—among HCWs underscores the need for workplace-centered interventions. Structured health promotion programs, including on-site fitness facilities, nutrition education, and scheduled protected meal breaks, could be especially beneficial. Moreover, given that many respondents expressed a willingness to adopt healthier behaviors, institutional support may bridge the gap between intention and sustained lifestyle change. As HCWs serve as role models for patient populations, investing in their well-being not only improves individual health outcomes but also has the potential to reinforce public health messaging through more credible, lived experiences.

From this perspective, it is easy to understand how effective strategies in matters of health promotion and prevention can bring significant benefits for optimal management of resources (19), both human and economic, as well as an improvement in terms of effectiveness, efficiency and performance of the Italian healthcare system.

## 5 Conclusion

In this observational study, we analyzed the characteristics of HCWs, focusing on the prevalence of the most common risk factors predisposing for NCDs and it was observed that their distribution changes significantly based on the sex, age and profession of the individual subject. It is necessary, however, to underline that the data collected regarding the correlation of these with shift work, both in this study and at an international level are still few and inconclusive. For this reason, it is still difficult to make a significant comparison of the association of risk factors with the different professional categories and respective shifts. However, from the analysis it emerges that, despite greater awareness compared to the general population, a large part of workers in the healthcare sector do not adhere to primary prevention strategies for NCDs and therefore they do not implement behaviors which can limit the risk of these pathologies. The data collected is currently insufficient and requires further investigation to clarify the correlation between these NCDs and shift work. Aside from the limitations, this is the first study of this type conducted at the University Hospital “P. Giaccone” and aims to provide a starting point to stimulate research on a larger scale. In conclusion, the prevalence of risk factors for NCD among healthcare workers require greater attention to ensure *ad hoc* prevention strategies, which include simple but highly effective actions, such as creating greater awareness and educating the population of reference to guarantee a high-quality health service, which cannot ignore a good state of physical, psychological and social health of those called upon to provide it.

## Data Availability

The raw data supporting the conclusions of this article will be made available by the authors, without undue reservation.
